# Short-course antidepressant therapy reduces discontinuation syndrome while maintaining treatment efficacy in patients with refractory functional dyspepsia: A randomized controlled trial

**DOI:** 10.3389/fpsyt.2022.1063722

**Published:** 2022-12-08

**Authors:** Qian-Qian Wang, Li Cheng, Bi-Yu Wu, Ping Xu, Hong-Yi Qiu, Bo Wang, Xiu-Juan Yan, Sheng-Liang Chen

**Affiliations:** Division of Gastroenterology and Hepatology, Key Laboratory of Gastroenterology and Hepatology, Ministry of Health, Shanghai Institute of Digestive Disease, Renji Hospital, School of Medicine, Shanghai Jiao Tong University, Shanghai, China

**Keywords:** neuromodulator, antidepressant discontinuation syndrome, psychosomatic disease, functional dyspepsia, flupentixol-melitracen

## Abstract

**Background and objective:**

Long-course (LC) antidepressants for the treatment of disorders of gut-brain interaction, such as refractory functional dyspepsia (rFD), pose patients at risk of antidepressant discontinuation syndrome (ADS). Short-course (SC) therapy of rapid-acting antidepressant may reduce discontinuation syndromes while maintaining efficacy for dyspeptic symptoms. However, the evidence-based research is lacking. This study aims to determine whether SC therapy with antidepressants could decrease the risk of ADS with comparable treatment efficacy to LC therapy in rFD.

**Methods:**

This randomized clinical trial with rFD patients was conducted at a tertiary hospital in China. Participants (*N* = 240) were randomly allocated to receive flupentixol-melitracen (FM) plus omeprazole therapy for 2 (SC group) or 4 (LC group) weeks, respectively. Scores for Leeds Dyspepsia Questionnaire (LDQ), Generalized Anxiety Disorder-7 (GAD-7) and Patient Health Questionnaire-9 for Depression (PHQ-9) were assessed at baseline and every 2 weeks, ending at 4 weeks after treatment. ADS was assessed after drug cessation. Medication possession ratio (MPR) for FM was calculated.

**Results:**

The severity and incidence of ADS of patients in SC group were significantly lower than those in LC group (0.60 ± 0.62 vs. 1.71 ± 1.58 and 3.64 vs. 39.45%; both *P* < 0.0001). The MPR values for FM were significantly higher in patients of SC group than in LC group (*P* < 0.0001). Scores for LDQ, GAD-7 and PHQ-9 decreased in patients of both groups, and the symptom improvement in SC group was comparable to that in LC group after treatment.

**Conclusions:**

Compared to 4-week FM therapy, the 2-week FM therapy reduces the risk of ADS with non-inferior treatment efficacy in patients with rFD.

**Clinical trial registration:**

Clinical trials.gov, identifier NCT05099913.

## Introduction

Functional dyspepsia (FD) affects up to 16% of the general population, and is characterized by abdominal symptoms including postprandial fullness, early satiety, epigastric pain or burning, which are unexplained after a routine clinical examination (1). As one kind of disorders of gut-brain interaction (DGBI), psychological factors, such as mental stress and distress, may contribute to the development of FD symptoms (2, 3). Therefore, FD is regarded as a gut-associated psychosomatic disease due to the involvement of psychological factors in the pathogenesis of the disease (4). Current therapeutic options for FD include anti-acids, proton pump inhibitors (PPIs), prokinetics, eradication of Helicobacter pylori (*H. pylori*) and central neuromodulators (1). These medications can relieve symptoms in a subset of patients, while a considerable proportion of FD patients fail to achieve satisfactory effects with continuous symptoms over months. Patients who display persisting FD symptoms for over 6 months and are unresponsive to at least two medical treatments, such as antiacids, PPIs, prokinetics, and eradication of *H. pylori*, are defined as refractory FD (rFD) (5, 6). Central neuromodulators, such as tricyclic antidepressants (TCAs) and serotonin-norepinephrine reuptake inhibitors (SNRIs), have been shown to be beneficial and are recommended for the treatment of FD (7–9).

With the increasing use of neuromodulators in DGBI, side effects associated with neuromodulators have emerged, which are reported to be the most common barriers to neuromodulator use (10). Besides, currently used neuromodulators are mainly slow-acting agents requiring at least 6–8 weeks for their actions, which might lead to stigma attributed to antidepressant and treatment non-adherence (11). In addition to the side effects and antidepressant non-adherence, the clinical use of neuromodulators is further complicated by antidepressant discontinuation syndrome (ADS) (12). ADS is characterized by a series of symptoms originated from central nervous system (CNS), including **F**lu-like symptoms, **I**nsomnia, **N**ausea, **I**mbalance, **S**ensory disorders, and **H**yperarousal (anxiety or agitation), summarized as **FINISH** (13, 14). The discontinuation symptoms generally occur within 2–4 days after drug cessation or tapering and last for 1–2 weeks (occasionally up to 1 year) (12). The incidence of ADS ranges from 9 to 66% in different types of antidepressants (12, 13, 15–17). The underlying mechanisms of ADS are still unclear. It might be associated with elevated synaptic levels of serotonin (17, 18), as well as increased levels of neurotrophic factors (19–21), dopamine receptors (22), and pro-inflammatory cytokines (16, 23) in brain. Currently, the effectiveness and safety of different approaches to discontinuing antidepressants are still uncertain (24, 25). Therefore, the prevention and management of ADS is of great clinical significance and needs to be considered before initiating antidepressant therapy.

The literature suggests that tapering of drug is unnecessary after treatment with an antidepressant for <4 weeks, indicating a low risk of ADS after short-term application of antidepressants (12). ADS occurs in ~20% of patients who receive antidepressant treatment for 6 weeks or longer (15, 16). Based on these findings, we presume that short-course (SC) antidepressant therapy might not induce ADS, while long-course (LC, 4 weeks or longer) therapy could increase the risk of ADS. Currently, neuromodulators are generally applied for 4–12 weeks in FD in literature (7, 8, 11, 26), which could pose patients at a risk of ADS. Reducing the course of neuromodulators appears to be a viable way to prevent ADS in FD treatment. However, it may raise the concern about the insufficiency of therapeutic efficacy for FD.

Low to modest dosages of TCAs provide the most convincing evidence of benefits in treating chronic gastrointestinal (GI) pain and DGBI in Rome Foundation Working Team Report (3). Neuromodulators augmentation treatment (TCAs/SNRIs plus antipsychotics) is recommended by the Rome Committees for DGBI when monotherapy is unsuccessful or produces side effects (3). Flupentixol-melitracen (FM, Deanxit, H. Lundbeck A/S, Denmark), which contains TCA and antipsychotic agents, was reported to be the most widely used antidepressant for depression disorders or psychosomatic diseases in six major cities of China from 2013 to 2018 (27). Flupentixol, as an antipsychotic agent, acts directly on the dopamine D_1_ and D_2_ receptors on the presynaptic membrane and increases dopamine concentrations in the synaptic space (28, 29). Melitracen, as a TCA, can increase the concentration of norepinephrine (NE) and serotonin (5-HT) in the synaptic space (30). FM at small doses has shown benefits for patients with rFD in east China (31). Although FM has been used for 8 weeks in the treatment of FD in China and America (32, 33), as a rapid-acting agent, FM treatment for 2 weeks has been reported to significantly improve gastroesophageal reflux symptoms (34). Besides, it has been shown that FM relieves anxiety and depression symptoms within 2 weeks in chronic somatic diseases in a randomized clinical trial (35). Therefore, it is probable that FM treatment for 2 weeks could be effective in relieving FD symptoms while reducing the risk of ADS. However, evidence-based research is still lacking.

In this prospective randomized clinical trial, we compared ADS and treatment outcome in patients with rFD receiving SC or LC FM therapy. The study aimed to provide practical advice on the prevention of ADS in patients with rFD receiving neuromodulator treatment.

## Materials and methods

### Ethics

The study was conducted following the principles of Declaration of Helsinki and approved by the Ethics Committee of Renji Hospital (Approval No. 2015-038K). The clinical trial was registered in ClinicalTrials.gov (NCT05099913) (https://clinicaltrials.gov/). An informed consent form was obtained from each participant before initiation of study protocol. The potential adverse effects of medications were explained in the same way to the patients from both groups before research.

### Participants

In this randomized controlled trial, rFD patients with continuous symptoms over 6 months and unresponsive to at least two medications (histamine-2 blockers, PPIs, prokinetics, and *H. pylori*) were recruited at the outpatient clinic of Renji Hospital from October 2020 to February 2022. A total of 269 patients were enrolled and assessed for eligibility by experienced gastroenterologists. The inclusion criteria were: (i) met the Rome IV criteria for FD; (ii) absence of abnormalities on physical examination, laboratory tests (including a routine blood test, blood glucose, and liver function examination), abdominal imaging or GI endoscopy; and the absence of H. pylori infection; (iii) without the use of sedative-hypnotic, antidepression, or antianxiety drugs in the past 6 months; and (iv) signed the informed consent of this study. The exclusion criteria were: (i) with organic diseases that might impair gastric function such as peptic ulcer, GI cancer, diabetes, scleroderma, nervous system disease, severe liver disease, heart disease, or alcoholism disorders; (ii) pregnancy or lactation; (iii) difficulty completing follow up or various factors affecting compliance; or (iv) a history of allergic reaction to any of the drugs used in the study.

### Study design and interventions

The study was a prospective, randomized, controlled clinical trial. Eligible patients were evaluated by experienced gastroenterologists, and then introduced with the study protocol. All the enrolled patients were randomly divided into two groups by an independent investigator using a computer-generated random number table. The statistician was blinded to treatment allocation.

Different medication regimens were applied in two groups. Patients in SC group received 560 mg omeprazole (20 mg, bid) and 147 mg FM (1 tablet, containing flupentixol 0.5 mg and melitracen 10 mg, qd) with a 2-week treatment course. Patients in LC group received 560 mg omeprazole (20 mg, bid) and 294 mg FM (1 tablet, qd) with a 4-week treatment course. Omeprazole was applied twice daily before breakfast and supper. FM was applied once daily before breakfast. We didn't distinguish epigastric pain syndrome or postprandial distress syndrome of FD symptoms in our research as many patients with rFD displayed overlapping symptoms.

Patients in both groups received education about disease and healthy lifestyle before initiation of antidepressant. The education was provided by experienced gastroenterologists through clinician-patient communication. It could establish patients' understanding of the disease and alleviate their concerns about the drug, which has been reported in our previous research (36): (1) Brain is the “controller” of GI tract, and the FD symptoms are caused by disorders of gut-brain interaction, rather than a sole GI or psychological problem; (2) Neuromodulators are used to relieve FD symptoms through regulating functions of GI tract and brain, which mainly acts on peripheral nervous system; (3) Neuromodulators are used in FD at lower doses and shorter courses, which is different from psychiatry patients. Lifestyle suggestions were as follows: (1) Maintain regular sleep (37); (2) Keep regular meal patterns, and eat a balanced low-fat diet with more vegetables and fruits (38–40); (3) Recommend aerobic physical exercise for 150–300 min per week (41). These lifestyle suggestions were also provided as a take-home note to the patients.

### Outcome measurements

The primary endpoint was the proportion of patients with ADS within 2 weeks upon drug cessation at the clinic, according to the FINISH symptoms (13, 14). The secondary endpoints included treatment outcomes at the end of treatment and 4-week follow-up period, as well as medication adherence. Treatment outcomes of dyspeptic symptoms (42, 43) and psychological status (44, 45) were assessed by self-reporting questionnaires at baseline, and every 2 weeks during treatment, ending at 4 weeks after treatment.

The FINISH questionnaire consisted of six items, to assess severity of discontinuation symptoms associated with antidepressants: (1) **F**lu-like symptoms; (2) **I**nsomnia: difficulty falling asleep or waking up; (3) **N**ausea (vomiting occasionally); (4) **I**mbalance (extrapyramidal symptoms): limb tremor and dyskinesia; (5) **S**ensory disturbances: itch, numbness, shock-like, palpitation, sweating abnormalities, or abdominal discomfort; (6) **H**yperarousal and other emotional distress: anxiety, agitation, or depression (14, 46, 47). Each item was ranked on a 4-point scale and the scoring criteria was as follows: 0, minimal, negligible; 1, mild, without affecting daily life; 2, moderate, affecting daily life but not interrupting ongoing activities; 3, severe, interrupting daily activities. The final FINISH score was calculated as the sum of the six items. Higher score indicated more severe symptoms. Considering the complication and vagueness of symptoms, scores ≥2 were defined as ADS (when the symptoms affected patients' daily life or at least two kinds of discontinuation symptoms occurred).

Leeds Dyspepsia Questionnaire (LDQ) (mandarin version) was applied to assess frequency and severity of dyspeptic symptoms, including epigastric pain, epigastric burning, postprandial fullness, early satiety, acid reflux, nausea, belch, and vomiting (43). The LDQ scores of 0–4 were classified as very mild dyspepsia, 5–8 as mild dyspepsia, 9–15 as moderate dyspepsia, and >15 as severe or very severe dyspepsia. Overall resolution of dyspeptic symptoms was defined as over 50% reduction in LDQ score.

Generalized Anxiety Disorder Scale (GAD-7) was applied to screen generalized anxiety disorder (44). It consisted of seven items and each item had 4 ranks (0–3). The final GAD-7 scores were calculated as the sum of the seven items. The GAD-7 scores of 0–4 were classified as the absence of anxiety, 5–9 as mild, 10–14 as moderate, and ≥15 as severe. The Patient Health Questionaire-9 Depression Scale (PHQ-9) was applied to screen depression disorders consisting nine items (45). Each item had 4 ranks (0–3). The final PHQ-9 scores were calculated as the sum of the nine items. The PHQ-9 scores of 0–4 were classified as none or minimal depression, 5–9 as mild, 10–14 as moderate, 15-19 as moderately severe, and ≥20 as severe depression.

Medication compliance was evaluated by calculating the medication possession ratio (MPR) (48). MPR was defined as the number of drugs taken by the patient relative to the amount prescribed during treatment (26). The MPR value of each patient was recorded after treatment.

### Statistical analysis

The sample size was calculated assuming a 15% difference of ADS incidence between two groups (SC was 0.05 and LC was 0.2) (16), with a significance level of 0.05 (α) and a power of 95% (1 – β) in the two-sided test. Additionally, the total sample size was 240 subjects with 108 patients per group considering ~10% of withdrawing or loss to follow-up.

Drug efficacy, treatment compliance and the incidence of ADS were analyzed based on the per-protocol population. For the primary endpoint, the proportion of patients with ADS within 2 weeks after drug cessation, according to the FINISH score, was calculated in the per-protocol set. Chi-square test and Fisher's exact tests were used to compare the primary endpoint, the proportion of patients with overall alleviation and secondary endpoints between two groups. Paired *t*-test was applied to compare the difference in symptom score of each group before and after treatment. Unpaired *t*-test was used to compare the difference of symptom score and medication compliance between two groups. Pearson's correlation analysis was used to determine the correlation of psychological status and dyspeptic symptoms. Continuous variables were displayed as mean ± SD. The data were analyzed using the SPSS 24.0 (IBM, Armonk, NY, USA). Statistical significance for all analysis was determined at the *P* < 0.05 level.

## Results

### Demographics and baseline clinical characteristics

**Figure 1:**
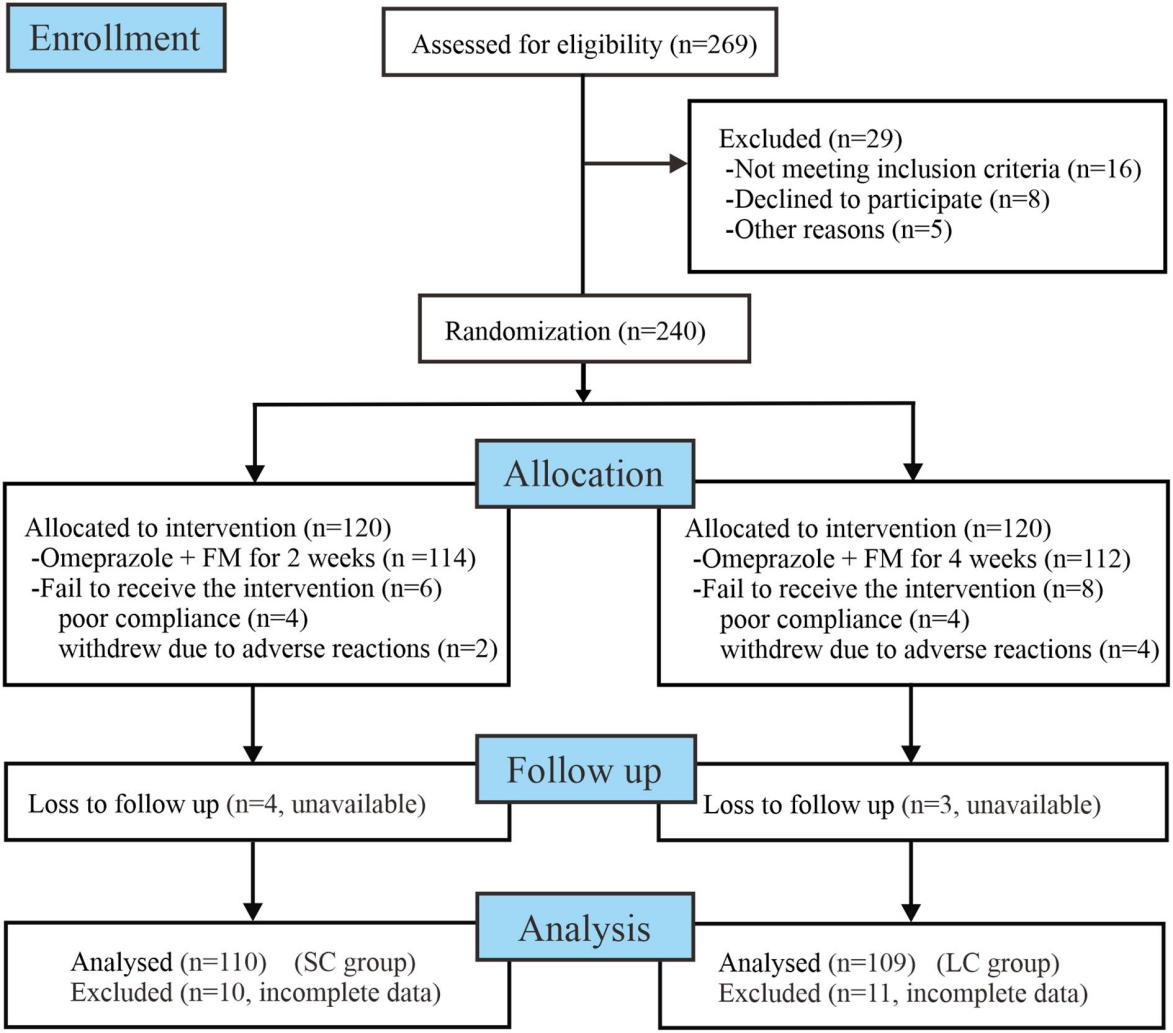
Workflow of randomization and treatment procedures of enrolled patients. FM, flupentixol-melitracen; SC, short-course; LC, long-course.

The workflow of randomization and treatment was displayed in Figure 1, following the CONSORT 2010 guidelines (http://www.consort-statement.org/consort-2010). In this study, 269 enrolled patients were assessed for eligibility for the research. Amongst them, 16 did not meet the inclusion criteria, eight declined to participate the research, and five did not complete the whole follow-up process due to individual reasons. In total, 240 patients diagnosed with FD were enrolled into our study and initiated antidepressant treatment. These patients were randomly allocated to receive 2- or 4-week FM treatment combined with omeprazole. During the research process, 20 patients were excluded from the program due to protocol violations, adverse effects, or loss to follow-up. Finally, 220 patients (110 patients of SC group, 109 patients of LC group) who completed the treatment and follow-up procedures were included into data analysis (per-protocol analysis). The demographic characteristics, baseline dyspeptic symptom scores, anxiety and depression scores of enrolled patients were shown in Table 1. Before treatment, two groups of patients were well-balanced in gender, age, duration of disease, and body mass index (BMI). There was no significant difference in baseline dyspeptic symptom scores, anxiety, and depression scores between two groups.

**Table 1:** Demographic and baseline clinical characteristics of the enrolled patients with refractory functional dyspepsia in per-protocol analysis.

**Characteristics**	**SC group**	**LC group**	***P*-value**
*n*	110	109	/
Sex ratio (F/M)	1.89	1.60	0.54
Age (years)	42.19 ± 11.26	42.85 ± 11.82	0.65
Disease duration (months)	10.74 ± 3.13	10.70 ± 3.43	0.93
BMI (kg/m^2^)	21.68 ± 2.29	21.51± 1.62	0.52
LDQ scores	8.66 ± 2.02	8.69 ± 2.77	0.92
GAD-7 scores	8.97 ± 2.67	9.00 ± 2.55	0.94
PHQ-9 scores	5.96 ± 3.14	5.94 ± 2.56	0.94

Values are presented as mean ± SD. SC, short-course; LC, long-course; BMI, body mass index; LDQ, Leeds Dyspepsia Questionnaire; GAD-7, Generalized Anxiety Disorder Scale; PHQ-9, Patient Health Questionnaire Depression Scale.

### ADS

**Figure 2:**
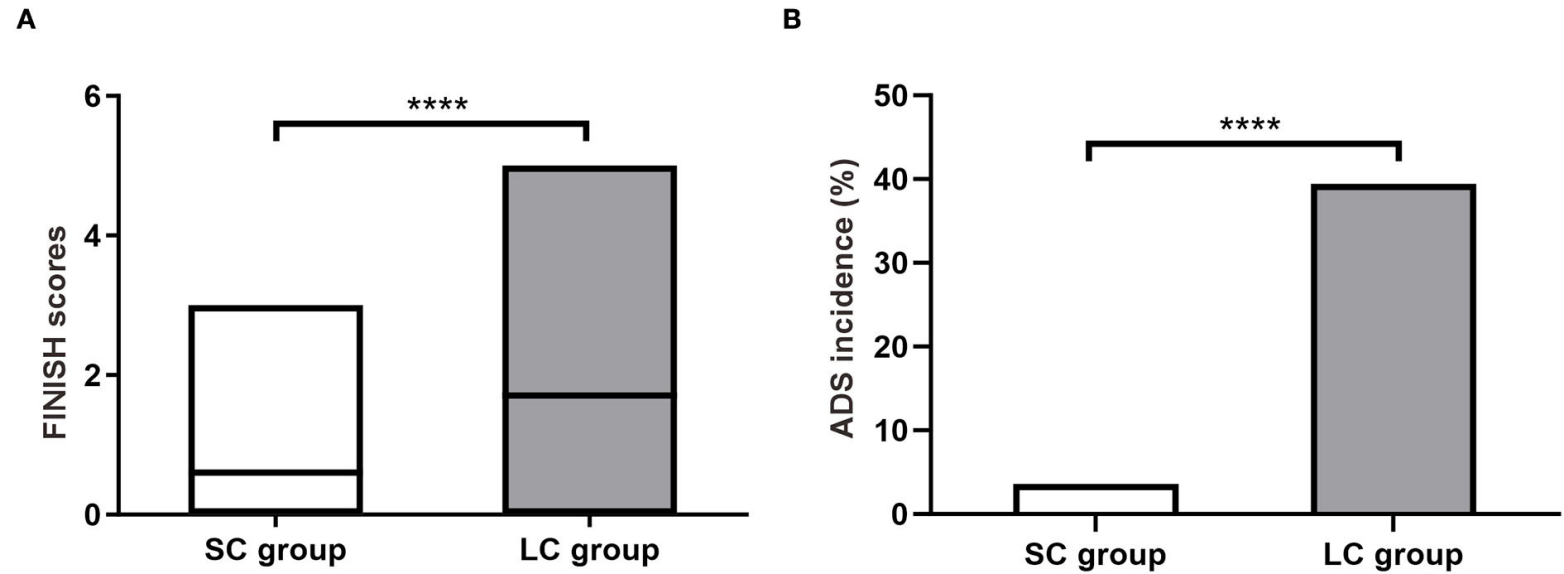
Antidepressant discontinuation syndrome (ADS) in patients with refractory functional dyspepsia. **(A)** The FINISH scores in patients of SC group (*n* = 110) and LC group (*n* = 109). **(B)** Incidence of ADS after drug discontinuation. SC, short-course; LC, long-course. ****P < 0.0001.

Discontinuation symptoms were assessed within 2 weeks after drug cessation. The FINISH questionnaire showed that most symptoms were mild with scores ranging from 0 to 5 (Figure 2A). The average FINISH score in SC group was significantly lower than that in LC group (0.60 ± 0.62 vs. 1.71 ± 1.58, *P* < 0.0001; Figure 2A). Among these patients, the incidence of ADS (FINISH score ≥ 2) in SC group was significantly lower than that in LC group [3.64% (4/110) vs. 39.45% (43/109), *P* < 0.0001; Figure 2B]. Patients receiving 2-week FM treatment had a significantly lower risk of ADS compared to patients receiving 4-week FM treatment (Relative risk 9.75, 95% CI 3.84–25.57). These results indicated that as the duration of treatment increased from 2 to 4 weeks, patients would face a remarkably increased relative risk of ADS. Besides, the discontinuation symptoms would become more complicated and serious.

In our research, discontinuation symptoms generally occurred within 3 days after drug cessation, and most symptoms were overlapping, as shown in Table 2. Flu-like symptoms and nausea were not observed in our research. Insomnia and emotional distress were the most common discontinuation symptoms. The incidence of insomnia in SC group was significantly lower than that in LC group (2.73 vs. 34.86%, *P* < 0.0001). The incidence of hyperarousal in SC group was also significantly lower than that in LC group (0.91 vs. 16.51%, *P* < 0.0001). In LC group, 9.17% of patients reported imbalance (tremor and dyskinesia), which was significantly higher than that in SC group (9.17 vs. 0.91%, *P* < 0.01). In LC group, 6.42% of patients developed sensory disturbance (sweating abnormalities and epigastric discomfort), while only 0.91% of patients in SC group reported heavy sweating. The incidences of different discontinuation symptoms were significantly higher in LC group than that in SC group (*P* < 0.01 or *P* < 0.0001; Table 2). Most discontinuation symptoms relieved spontaneously within 2 weeks during follow-up.

**Table 2:** Symptoms of antidepressant discontinuation syndrome in patients with refractory functional dyspepsia.

**Symptoms**	**SC group (*n* = 110)**	**LC group (*n* = 109)**
Insomnia	3 (2.73)	38 (34.86)****
Imbalance^†^	1 (0.91)	10 (9.17)**
Sensory disturbances^‡^	1 (0.91)	7 (6.42)
Hyperarousal^§^	1 (0.91)	18 (16.51)****

† Imbalance included limb tremor and dyskinesia.

‡ Sensory disturbances included sweating abnormalities and abdominal discomfort.

§ Hyperarousal included anxiety and agitation.

SC, short-course; LC, long-course. **P < 0.01, ****P < 0.0001 (χ2 test).

Data are presented as *n* (%).

### Treatment outcomes of dyspeptic symptoms and psychological condition

**Figure 3:**
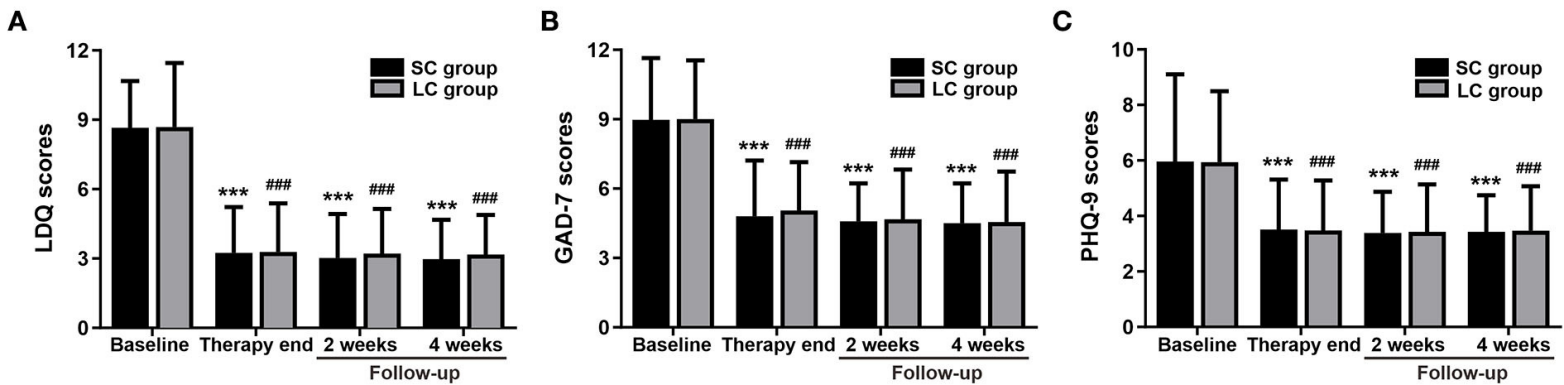
Treatment outcomes of patients with refractory functional dyspepsia receiving 2- or 4-week FM treatment. **(A)** LDQ scores, **(B)** GAD-7 scores, and **(C)** PHQ-9 scores in SC group (*n* = 110) and LC group (*n* = 109). SC, short-course; LC, long-course; LDQ, Leeds Dyspepsia Questionnaire; GAD-7, Generalized Anxiety Disorder-7; PHQ-9, Patient Health Questionnaire-9 Depression Scale. ***P < 0.001, comparison with baseline in SC group; ^###^*P* < 0.001, comparison with baseline in LC group.

Patients in SC group and LC group were treated with 2- and 4-week FM medication, respectively, then they were followed up for 4 weeks after treatment. Most patients showed quick response to the therapy and reported symptom relief after 2–3 days. After treatment, the LDQ scores in SC group and LC group decreased compared to baseline, respectively (*P* < 0.001; Figure 3A), with no significant difference between two groups (SC 3.25 ± 1.98 vs. LC 3.28 ± 2.10, *P* = 0.89; Figure 3A). The patients were followed up at 2 and 4 weeks after completing therapy, and the reductions of LDQ scores in SC group was slightly higher than in LC group while showing no significant difference between two groups (2-week, SC 3.03 ± 1.89 vs. LC 3.21 ± 1.93, *P* = 0.48; 4-week, 2.97 ± 1.71 vs. 3.17 ± 1.72, *P* = 0.38; Figure 3A). It showed that the improvements of dyspeptic symptoms could be maintained for 4 weeks after treatment in both groups, and FM treatment for 2 or 4 weeks had no significant difference in relieving dyspeptic symptoms. The treatment efficacy for dyspeptic symptoms was shown in Table 3. Patients showing over 50% reduction in LDQ scores after treatment were considered as effective treatment response. There was no significant difference in the efficacy rate between two groups at the end of treatment (SC 83.64 vs. LC 81.65%, *P* = 0.85). During follow-up, the treatment efficacy showed no significant difference between the two groups (2-week, 80.91 vs. 72.48%, *P* = 0.24; 4-week, 78.18 vs. 71.56%, *P* = 0.41). The therapeutic effect could be maintained for 4 weeks after completion of treatment and recurrence of FD symptoms was not observed in both groups.

**Table 3:** Effectiveness of dyspeptic symptoms during follow-up.

**Effectiveness^†^**	**SC group (*n* = 110)**	**LC group (*n* = 109)**	***P*-value**
Therapy end	92 (83.64)	89 (81.65)	0.85
2-week follow-up	89 (80.91)	79 (72.48)	0.24
4-week follow-up	86 (78.18)	78 (71.56)	0.41

† Over 50% reduction in LDQ score was considered as effective treatment response.

Data were presented as *n* (%).

Anxiety status was shown in Figure 3B. After treatment, the GAD-7 scores decreased significantly in SC group and LC group compared to baseline, respectively (*P* < 0.001; Figure 3B), with no significant difference between two groups (SC 4.81 ± 2.41 vs. LC 5.06 ± 2.09, *P* = 0.42). The reductions of GAD-7 scores sustained for at least 4 weeks during follow-up and showed no significant difference between two groups (2-week, SC 4.58 ± 1.64 vs. LC 4.68 ± 2.15, *P* = 0.71; 4-week, 4.51 ± 1.72 vs. 4.56 ± 2.18, *P* = 0.85; Figure 3B). It revealed that the improvements of anxiety scores could sustain for at least 4 weeks after treatment in both groups. These results suggested that 2 or 4 weeks of FM treatment had no significant difference in improving anxiety symptoms.

Depression status was shown in Figure 3C. The PHQ-9 scores significantly decreased in SC group and LC group compared to baseline, respectively (*P* < 0.001; Figure 3C), with no significant difference between two groups (3.51 ± 1.80 vs. 3.48 ± 1.79, *P* = 0.90). The patients were followed up at 2 and 4 weeks after completing treatment, and the reduction of PHQ-9 scores persisted and showed no significant difference between two groups (2-week, 3.39 ± 1.48 vs. 3.43 ± 1.71, *P* = 0.85; 4-week, 3.43 ± 1.32 vs. 3.47 ± 1.60, *P* = 0.84; Figure 3C). It revealed that the improvements of depressive emotions could be maintained for 4 weeks after completion of treatment in both groups, and FM treatment for 2 or 4 weeks of FM treatment had no significant difference in relieving depressive condition.

### Correlation analysis between dyspeptic symptoms and psychological condition

**Figure 4:**
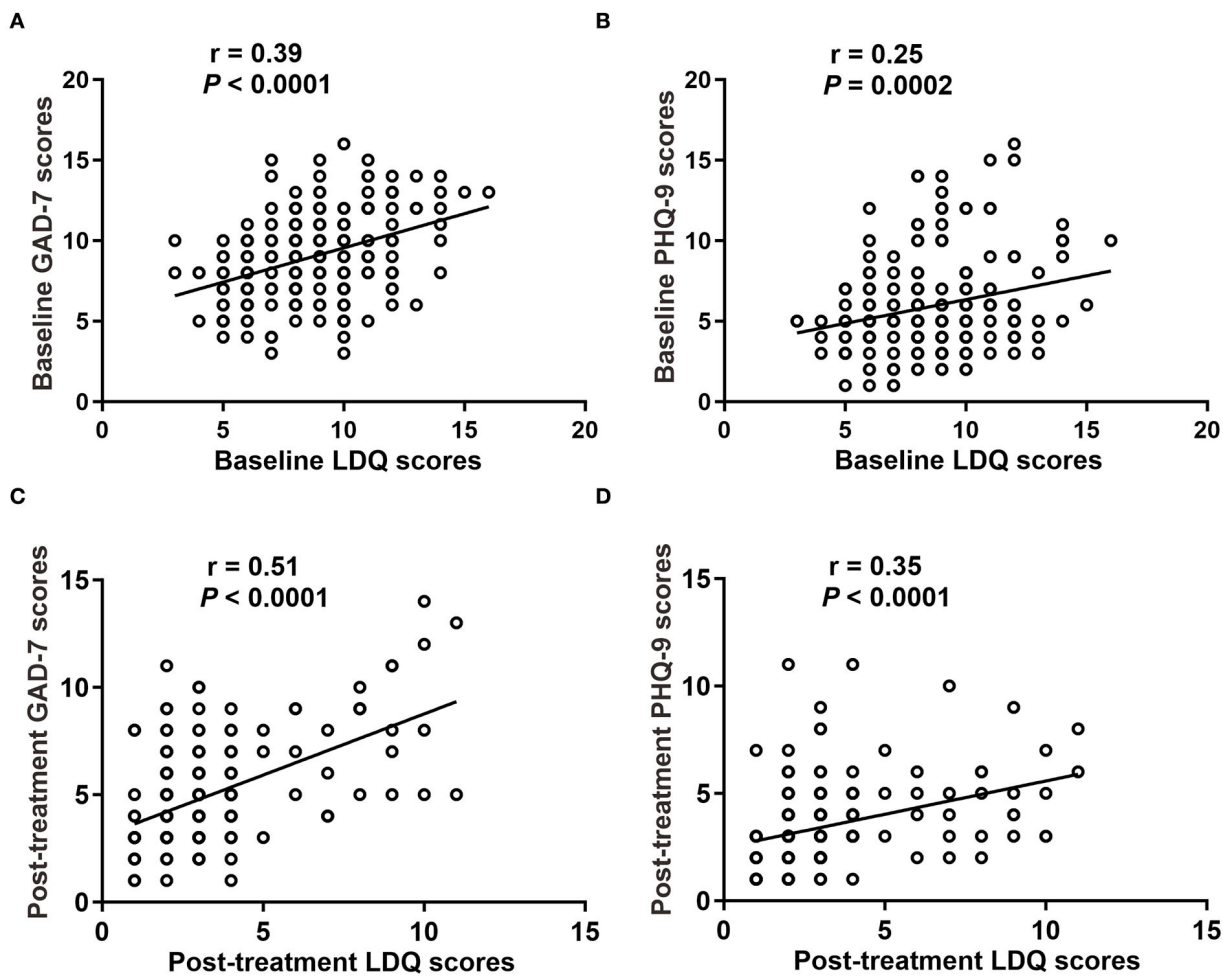
Correlation between dyspepsia symptoms with emotional disorders. **(A)** Baseline GAD-7 scores with LDQ scores. **(B)** Baseline PHQ-9 scores with LDQ scores. **(C)** Post-treatment GAD-7 scores with LDQ scores. **(D)** Post-treatment PHQ-9 scores with LDQ scores. LDQ, Leeds Dyspepsia Questionnaire; GAD-7, Generalized Anxiety Disorder-7; PHQ-9, Patient Health Questionnaire-9 Depression Scale.

We analyzed the correlation between LDQ scores with GAD-7 or PHQ-9 scores in two groups. The results revealed that patients with rFD were usually accompanied by emotional distress (Figure 4). It showed that LDQ score was positively correlated with GAD-7 score (*r* = 0.398, *P* < 0.001; Figure 4A) and PHQ-9 score (*r* = 0.247, *P* < 0.001; Figure 4B) before treatment. After treatment, dyspeptic symptoms and emotional disorders were significantly decreased. Post-treatment LDQ score was also positively correlated with GAD-7 score (*r* = 0.498, *P* < 0.001; Figure 4C) and PHQ-9 score (*r* = 0.349, *P* < 0.001; Figure 4D). These results indicated that there was a correlation between dyspeptic symptoms with emotional distress in rFD.

### Medication compliance

**Figure 5:**
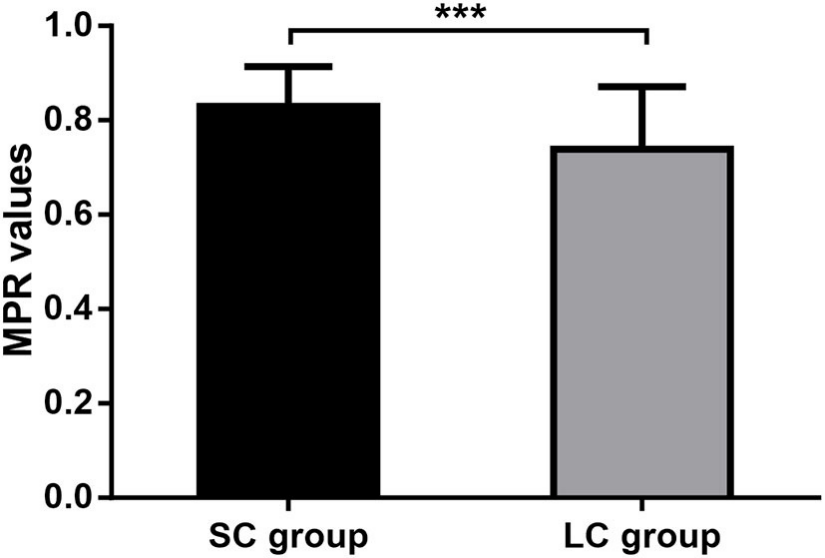
Medication adherence. MPR, medication possession ratio; SC, short-course; LC, long-course; ***P < 0.001.

We also evaluated the medication compliance for FM in patients of both groups. It showed that patients treated with FM for 2 weeks had an average MPR value of 0.83 ± 0.08, while those treated with FM for 4 weeks had an average MPR value of 0.74 ± 0.13. Medication compliance for FM was significantly higher in SC group than in LC group (*P* < 0.0001; Figure 5). It suggested that longer treatment course may affect the patient's medication compliance.

### Safety analyses

In total, five patients (5/110, 4.54%) in SC group and twenty-two patients (22/109, 20.18%) in LC group reported adverse effects during treatment, including dry mouth, sleep disturbance, tardive dyskinesia, and increased heart rate (Table 4). Dry mouth was found in 5 patients (4.55%) of SC group and thirteen patients (11.93%) of LC group. Four patients (3.64%) of SC group and twenty patients (18.35%) of LC group reported sleep disturbance. The incidence was significantly lower in SC group than LC group (3.64 vs. 18.35%, *P* < 0.05; Table 4). Tardive dyskinesia was found in eight patients (7.34%) of LC group and one patient (0.91%) of SC group, and it was significantly lower in SC group than in LC group (0.91 vs. 7.34%, *P* < 0.01; Table 4). One patient (0.91%) in SC group and three patients (2.75%) in LC group reported increased heart rate. All these adverse effects were mild. No severe events occurred in the research.

**Table 4:** Adverse effects during treatment.

**Adverse effects**	**SC group (*n* = 110)**	**LC group (*n* = 109)**
Dry mouth	5 (4.55)	13 (11.93)
Sleep disturbance	4 (3.64)	20 (18.35)**
Tardive dyskinesia	1 (0.91)	8 (7.34)*
Increased heart rate	1 (0.91)	3 (2.75)

SC, short-course; LC, long-course; *P < 0.05; **P < 0.01 (χ2 test).

Data were presented as *n* (%).

## Discussion

Neuromodulators could attenuate visceral nociception and exert profound effects on peripheral nervous system (e.g., GI tract), and has been proven to be effective in the treatment of rFD (6). However, concerns about side effects and ADS were the most common barriers to neuromodulator use (10, 13). Gastroenterologists are concerned about ADS and side effects of LC therapy and the insufficiency of efficacy of SC therapy in rFD. Therefore, our research compared ADS, dyspeptic symptoms and side effects in patients with rFD receiving SC neuromodulator therapy vs. those receiving LC therapy. The results showed that 2-week FM treatment obtained comparable efficacy to 4-week FM treatment with fewer ADS and adverse effects in patients with rFD. This research could provide practical advice for gastroenterologists when treating FD patients who are worried about antidepressant dependence (11, 36).

ADS is one of the most concerned issues in the clinical application of neuromodulators on DGBI. Longer treatment course may increase the risk of ADS and antidepressant therapy for <4 weeks is unlikely to induce ADS after drug discontinuation (12, 13, 15, 16, 46). Consistently, our research found that discontinuation symptoms were more severe in LC group than in SC group. The average FINISH score in SC group was significantly lower than that in LC group. Moreover, incidence of ADS (FINISH score ≥ 2) in SC group was very low (3.64%) while it was remarkably higher in LC group (39.45%). The incidence of individual discontinuation symptoms (including insomnia, imbalance, sensory disturbances and hyperarousal) was also lower in SC group (0.91–2.73%) than in LC group (6.42–34.86%). These results indicated that shortening the course of neuromodulator therapy may reduce ADS.

The mechanism of ADS has not been fully illustrated yet (12, 15, 16). The oppositional model of tolerance explained that the drug effect on serotonin function occurred at 2–4 weeks after treatment, with adaptive changes of serotonin actions in the brain (49). Neuromodulators act through increasing the concentration of neurotransmitters in the synaptic cleft, finally trigger synapse redistribution in terms of the number and types of receptors in the presynaptic and postsynaptic membranes. Afterwards, the biological balance of the neurotransmitter-receptor axis can be maintained by a continuous supply of neuromodulators (50, 51). After longer treatment courses, further adaptive changes in the brain may occur through serotonin receptors and transporters or other neurotransmitters (52, 53). The remodeling process requires at least 2–6 weeks for the altered neurotransmitters and membrane receptors to achieve the functional homeostasis (19, 28, 54–57). Therefore, it's unlikely to induce ADS before the remodeling process is completed, which could be the reason for low incidence of ADS in 2-week FM treatment group.

The treatment efficacy for FD symptoms in SC group was comparable to LC group in our research. In addition, the medication compliance for FM in LC group was significantly lower than that in SC group, perhaps it was due to the stigma associated with disease and antidepressants, which could hinder the treatment compliance and efficacy (11, 58). It seemed that 2-week treatment with good drug compliance is fairly sufficient to relieve FD symptoms. Moreover, most discontinuation symptoms disappeared within 2 weeks and the efficacy could be maintained for 4 weeks after drug cessation. No significant relapse of symptoms was observed during follow-up in both groups. These results also suggested that application of quick-responding neuromodulator FM for 2 weeks might relieve rFD symptoms with steady efficacy. Several patients in LC group reported anxiety overlapping abdominal discomfort during follow-up and these symptoms disappeared at the end of research, which might be caused by ADS and need to be distinguished from recurrence. Generally, the recurrence of symptoms usually took more than a few days to appear, and the reintroduction of antidepressants was needed to relieve the symptoms. Discontinuation symptoms occurred within 2–4 days upon drug cessation, and most symptoms were self-limiting and disappeared after 1–2 weeks (12, 13, 15). It seemed that 2-week FM treatment could achieve non-inferior effectiveness to 4-week FM treatment, meanwhile it could reduce the risk of ADS.

We also found an interesting phenomenon that there was a correlation between dyspeptic symptoms and emotional distress in patients with rFD. As is known to all, psychological morbidity is common in patients with DGBI (2). Patients' stigma and wrong perceptions of medications and disease could hinder treatment efficacy (58). It has been reported that effective communication of the rationale of the brain-gut axis, benefits of neuromodulators or behavioral health treatments could improve patient satisfaction and clinical outcomes (33, 36, 58, 59). Behavioral therapy, such as maintaining a low-fat diet, regular sleep, and moderate exercise, was reported to be beneficial in relieving FD symptoms (37–40). Therefore, our research also included patient education in the management of rFD, and these measures may contribute to achieving optimal therapeutic effect.

This research reveals for the first time about the viable way for the prevention of ADS in patients with FD. It shows that a 2-week FM treatment can prevent ADS with comparable therapeutic effect to a 4-week FM treatment, and it is relatively safe with less adverse effects in patients with rFD. It also provides a practical and worthwhile solution for gastroenterologists in the treatment of FD patients who are worried about ADS. Moreover, currently used neuromodulators in DGBI are mainly slow-acting and single-target drugs, which require at least 4 weeks of continuous treatment for therapeutic effects, while FM for 2 weeks could produce considerable efficacy in relieving gastroesophageal reflux symptoms and FD symptoms (34). As a multi-target and fast-acting neuromodulator, FM could provide a new perspective for the treatment of DGBI (54).

This research also has several limitations. Firstly, there is no controlled study of different types of neuromodulators. The drug onset time and mechanisms of neuromodulators may be different, which may be influential factors of ADS (12, 13, 15). Secondly, FM was previously used in America (32). Due to its fast onset, FM is the most widely used antidepressant for psychosomatic diseases in China, while the data of evidence-based research in other countries is limited (27, 31, 35). Thirdly, we didn't distinguish epigastric pain syndrome or postprandial distress syndrome in our research as many patients with rFD displayed overlapping symptoms. Lastly, the follow-up period after drug cessation is 4 weeks in the research, which may be insufficient to prove that the 2-week therapy could reduce recurrences of FD, and longer observation period is required for the assessment of recurrences in further studies.

## Conclusion

In summary, 2-week FM therapy combined with patient education can reduce ADS with comparable effectiveness to 4-week FM therapy in relieving dyspeptic symptoms and emotional distress of patients with rFD.

## Data availability statement

The original contributions presented in the study are included in the article/supplementary material, further inquiries can be directed to the corresponding authors.

## Ethics statement

The studies involving human participants were reviewed and approved by Ethics Committee of Renji Hospital (Approval No. 2015-038K). The patients/participants provided their written informed consent to participate in this study.

## Author contributions

S-LC designed the study. S-LC, X-JY, PX, and H-YQ performed the study. Q-QW and LC collected the data. B-YW and BW analyzed the data. Q-QW wrote the manuscript. S-LC and X-JY made critical revisions of the manuscript. All authors contributed to the conception and performance of research protocol. All authors approved the final manuscript.

## References

[1] Ford AC Mahadeva S Carbone MF Lacy BE Talley NJ. Functional dyspepsia. Lancet. (2020) 396:1689–702. 10.1016/S0140-6736(20)30469-4 33049222

[2] Drossman DA Tack J Ford AC Szigethy E Törnblom H Van Oudenhove L. Neuromodulators for functional gastrointestinal disorders (disorders of gut-brain interaction): A Rome Foundation Working Team Report. Gastroenterology. (2018) 154:1140–71. 10.1053/j.gastro.2017.11.279 29274869

[3] Black CJ Drossman DA Talley NJ Ruddy J Ford AC. Functional gastrointestinal disorders: advances in understanding and management. Lancet. (2020) 396:1664–74. 10.1016/S0140-6736(20)32115-2 33049221

[4] Bransfield RC Friedman KJ. Differentiating psychosomatic, somatopsychic, multisystem illnesses, and medical uncertainty. Healthcare. (2019) 7:114. 10.3390/healthcare7040114 31597359 PMC6955780

[5] Jiang SM Jia L Lei XG Xu M Wang SB Liu J. Incidence and psychological-behavioral characteristics of refractory functional dyspepsia: a large, multi-center, prospective investigation from China. World J Gastroenterol. (2015) 21:1932–7. 10.3748/wjg.v21.i6.1932 25684962 PMC4323473

[6] Cheong PK Ford AC Cheung CKY Ching JYL Chan Y Sung JJY. Low-dose imipramine for refractory functional dyspepsia: a randomised, double-blind, placebo-controlled trial. Lancet Gastroenterol Hepatol. (2018) 3:837–44. 10.1016/S2468-1253(18)30303-0 30361080

[7] Lacy BE Saito YA Camilleri M Bouras E DiBaise JK Herrick LM. Effects of antidepressants on gastric function in patients with functional dyspepsia. Am J Gastroenterol. (2018) 113:216–24. 10.1038/ajg.2017.458 29257140

[8] Ford AC Luthra P Tack J Boeckxstaens GE Moayyedi P Talley NJ. Efficacy of psychotropic drugs in functional dyspepsia: systematic review and meta-analysis. Gut. (2017) 66:411–20. 10.1136/gutjnl-2015-310721 26567029

[9] Keefer L Ko CW Ford AC. AGA Clinical practice update on management of chronic gastrointestinal pain in disorders of gut-brain interaction: expert review. Clin Gastroenterol Hepatol. (2021) 19:2481–8. 10.1016/j.cgh.2021.07.006 34229040

[10] Nulsen B LeBrett W Drossman DA Chang L. A survey of gastroenterologists in the United States on the use of central neuromodulators for treating irritable bowel syndrome. Aliment Pharmacol Ther. (2021) 54:281–91. 10.1111/apt.16467 34148256

[11] Yan XJ Luo QQ Qiu HY Ji CF Chen SL. The impact of stigma on medication adherence in patients with functional dyspepsia. Neurogastroenterol Motil. (2021) 33:e13956. 10.1111/nmo.13956 33184967

[12] Gabriel M Sharma V. Antidepressant discontinuation syndrome. CMAJ. (2017) 189:E747. 10.1503/cmaj.160991 28554948 PMC5449237

[13] Fava GA Cosci F. Understanding and managing withdrawal syndromes after discontinuation of antidepressant drugs. J Clin Psychiatry. (2019) 80:19com12794. 10.4088/JCP.19com12794 31774947

[14] Berber MJ. FINISH remembering the discontinuation syndrome. Flu-like symptoms, insomnia, nausea, imbalance, sensory disturbances, and hyperarousal (anxiety/agitation). J Clin Psychiatry. (1998) 59:255. 10.4088/JCP.v59n0509b 9632038

[15] Warner CH Bobo W Warner C Reid S Rachal J. Antidepressant discontinuation syndrome. Am Fam Phys. (2006) 74:449–56. 16913164

[16] Zabegalov KN Kolesnikova TO Khatsko SL Volgin AD Yakovlev OA Amstislavskaya TG. Understanding antidepressant discontinuation syndrome (ADS) through preclinical experimental models. Eur J Pharmacol. (2018) 829:129–40. 10.1016/j.ejphar.2018.04.003 29627310

[17] Fava GA Benasi G Lucente M Offidani E Cosci F Guidi J. Withdrawal symptoms after serotonin-noradrenaline reuptake inhibitor discontinuation: systematic review. Psychother Psychosom. (2018) 87:195–203. 10.1159/000491524 30016772

[18] Fava GA Bernardi M Tomba E Rafanelli C. Effects of gradual discontinuation of selective serotonin reuptake inhibitors in panic disorder with agoraphobia. Int J Neuropsychopharmacol. (2007) 10:835–8. 10.1017/S1461145706007462 17224089

[19] Yin XL Ma YY Liu YL Wang LX Du N Yang L. Changes of brain-derived neurotrophic factors in rats with generalized anxiety disorder before and after treatment. Eur Rev Med Pharmacol Sci. (2022) 26:1500–7. 35302194 10.26355/eurrev_202203_28214

[20] Paumier KL Sortwell CE Madhavan L Terpstra B Daley BF Collier TJ. Tricyclic antidepressant treatment evokes regional changes in neurotrophic factors over time within the intact and degenerating nigrostriatal system. Exp Neurol. (2015) 266:11–21. 10.1016/j.expneurol.2015.02.005 25681575 PMC4382385

[21] Kandil EA Abdelkader NF El-Sayeh BM Saleh S. Imipramine and amitriptyline ameliorate the rotenone model of Parkinson's disease in rats. Neuroscience. (2016) 332:26–37. 10.1016/j.neuroscience.2016.06.040 27365173

[22] Lammers CH Diaz J Schwartz JC Sokoloff P. Selective increase of dopamine D3 receptor gene expression as a common effect of chronic antidepressant treatments. Mol Psychiatry. (2000) 5:378–88. 10.1038/sj.mp.4000754 10889548

[23] Furtado M Katzman MA. Examining the role of neuroinflammation in major depression. Psychiatry Res. (2015) 229:27–36. 10.1016/j.psychres.2015.06.009 26187338

[24] Jauhar S Hayes J Goodwin GM Baldwin DS Cowen PJ Nutt DJ. Antidepressants, withdrawal, and addiction; where are we now? J Psychopharmacol. (2019) 33:655–9. 10.1177/0269881119845799 31111764 PMC7613097

[25] Van Leeuwen E van Driel ML Horowitz MA Kendrick T Donald M De Sutter AI. Approaches for discontinuation versus continuation of long-term antidepressant use for depressive and anxiety disorders in adults. Cochrane Database Syst Rev. (2021) 4:CD013495. 10.1002/14651858.CD013495.pub2 33886130 PMC8092632

[26] Wang B Luo QQ Li Q Cheng L Chen SL. Daily short message service reminders increase treatment compliance and efficacy in outpatients with functional dyspepsia: a prospective randomized controlled trial. J Gen Intern Med. (2020) 35:2925–31. 10.1007/s11606-020-06088-3 32779141 PMC7572925

[27] Yu Z Zhang J Zheng Y Yu L. Trends in antidepressant use and expenditure in six major cities in China from 2013 to 2018. Front Psychiatry. (2020) 11:551. 10.3389/fpsyt.2020.00551 32765307 PMC7378967

[28] Gottschling C Geissler M Springer G Wolf R Juckel G Faissner A. First and second generation antipsychotics differentially affect structural and functional properties of rat hippocampal neuron synapses. Neuroscience. (2016) 337:117–30. 10.1016/j.neuroscience.2016.08.055 27615033

[29] Juel C. Pre- and postsynaptic effects of dopamine antagonists on dopaminergic synaptic transmission in Helix pomatia. Comp Biochem Physiol C Comp Pharmacol Toxicol. (1983) 76:203–8. 10.1016/0742-8413(83)90064-6 6139250

[30] Chen X Cheng J Gong J. Deanxit can improve the dizziness, anxiety, and quality of life of patients with chronic subjective dizziness. Am J Transl Res. (2021) 13:9348−55. 34540052 PMC8430134

[31] Luo L Du L Shen J Cen M Dai N. Benefit of small dose antidepressants for functional dyspepsia: experience from a tertiary center in eastern China. Medicine. (2019) 98:e17501. 10.1097/MD.0000000000017501 31593119 PMC6799471

[32] Hashash JG Abdul-Baki H Azar C Elhajj II El Zahabi L Chaar HF. Clinical trial: a randomized controlled cross-over study of flupentixol + melitracen in functional dyspepsia. Aliment Pharmacol Ther. (2008) 27:1148–55. 10.1111/j.1365-2036.2008.03677.x 18331614

[33] Yan XJ Li WT Chen X Wang EM Liu Q Qiu HY. Effect of clinician-patient communication on compliance with flupentixol-melitracen in functional dyspepsia patients. World J Gastroenterol. (2015) 21:4652–9. 10.3748/wjg.v21.i15.4652 25914475 PMC4402313

[34] Yu YY Fang DC Fan LL Chang H Wu ZL Cao Y. Efficacy and safety of esomeprazole with flupentixol/melitracen in treating gastroesophageal reflux disease patients with emotional disorders. J Gastroenterol Hepatol. (2014) 29:1200–6. 10.1111/jgh.12552 24955450

[35] Wang L Zhong Z Hu J Rong X Liu J Xiao S. Sertraline plus deanxit to treat patients with depression and anxiety in chronic somatic diseases: a randomized controlled trial. BMC Psychiatry. (2015) 15:84. 10.1186/s12888-015-0449-2 25879863 PMC4403889

[36] Yan XJ Qiu HY Luo QQ Wang B Xu P Ji CF. Improving clinician-patient communication alleviates stigma in patients with functional dyspepsia receiving antidepressant treatment. J Neurogastroenterol Motil. (2022) 28:95–103. 10.5056/jnm20239 34980692 PMC8748843

[37] Li Y Gong Y Li Y He D Wu Y Wang H. Sleep disturbance and psychological distress are associated with functional dyspepsia based on Rome III criteria. BMC Psychiatry. (2018) 18:133. 10.1186/s12888-018-1720-0 29776354 PMC5960153

[38] Pesce M Cargiolli M Cassarano S Polese B De Conno B Aurino L. Diet and functional dyspepsia: clinical correlates and therapeutic perspectives. World J Gastroenterol. (2020) 26:456–65. 10.3748/wjg.v26.i5.456 32089623 PMC7015717

[39] Tabibian SR Hajhashemy Z Shaabani P Saneei P Keshteli AH Esmaillzadeh A. The relationship between fruit and vegetable intake with functional dyspepsia in adults. Neurogastroenterol Motil. (2021) 33:e14129. 10.1111/nmo.14129 33797127

[40] Duboc H Latrache S Nebunu N Coffin B. The role of diet in functional dyspepsia management. Front Psychiatry. (2020) 11:23. 10.3389/fpsyt.2020.00023 32116840 PMC7012988

[41] Yang YJ. An overview of current physical activity recommendations in primary care. Korean J Fam Med. (2019) 40:135–42. 10.4082/kjfm.19.0038 31122003 PMC6536904

[42] Moayyedi P Duffett S Braunholtz D Mason S Richards ID Dowell AC. The Leeds Dyspepsia Questionnaire: a valid tool for measuring the presence and severity of dyspepsia. Aliment Pharmacol Ther. (1998) 12:1257–62. 10.1046/j.1365-2036.1998.00404.x 9882035

[43] Leow HR Ching SM Sujarita R Mason S Richards ID Dowell AC. Mandarin version of the Leeds Dyspepsia Questionnaire: a valid instrument for assessing symptoms in Asians. J Dig Dis. (2014) 15:591–6. 10.1111/1751-2980.12183 25139629

[44] Herr NR Williams JW Benjamin S McDuffie J. Does this patient have generalized anxiety or panic disorder?: The Rational Clinical Examination systematic review. JAMA. (2014) 312:78–84. 10.1001/jama.2014.5950 25058220

[45] Wang W Bian Q Zhao Y Li X Wang W Du J. Reliability and validity of the Chinese version of the Patient Health Questionnaire (PHQ-9) in the general population. Gen Hosp Psychiatry. (2014) 36:539–44. 10.1016/j.genhosppsych.2014.05.021 25023953

[46] Henssler J Heinz A Brandt L Bschor T. Antidepressant withdrawal and rebound phenomena. Dtsch Arztebl Int. (2019) 116:355–61. 10.3238/arztebl.2019.0355 31288917 PMC6637660

[47] Jha MK Rush AJ Trivedi MH. When discontinuing SSRI antidepressants is a challenge: management tips. Am J Psychiatry. (2018) 175:1176–84. 10.1176/appi.ajp.2018.18060692 30501420

[48] Peterson AM Nau DP Cramer JA Benner J Gwadry-Sridhar F Nichol M. checklist for medication compliance and persistence studies using retrospective databases. Value Health. (2007) 10:3–12. 10.1111/j.1524-4733.2006.00139.x 17261111

[49] Fava GA Belaise C. Discontinuing antidepressant drugs: lesson from a failed trial and extensive clinical experience. Psychother Psychosom. (2018) 87:257–67. 10.1159/000492693 30149374

[50] Calabrese F Riva MA Molteni R. Synaptic alterations associated with depression and schizophrenia: potential as a therapeutic target. Expert Opin Ther Targets. (2016) 20:1195–207. 10.1080/14728222.2016.1188080 27167520

[51] Duman RS Aghajanian GK. Synaptic dysfunction in depression: potential therapeutic targets. Science. (2012) 338:68–72. 10.1126/science.1222939 23042884 PMC4424898

[52] Shapiro BB. Subtherapeutic doses of SSRI antidepressants demonstrate considerable serotonin transporter occupancy: implications for tapering SSRIs. Psychopharmacology. (2018) 235:2779–81. 10.1007/s00213-018-4995-4 30097698

[53] Coleman JA Green EM Gouaux E. X-ray structures and mechanism of the human serotonin transporter. Nature. (2016) 532:334–9. 10.1038/nature17629 27049939 PMC4898786

[54] Li YF. A hypothesis of monoamine (5-HT) - Glutamate/GABA long neural circuit: aiming for fast-onset antidepressant discovery. Pharmacol Ther. (2020) 208:107494. 10.1016/j.pharmthera.2020.107494 31991195

[55] Rafa-Zabłocka K Kreiner G Bagińska M Nalepa I. Selective Depletion of CREB in serotonergic neurons affects the upregulation of brain-derived neurotrophic factor evoked by chronic fluoxetine treatment. Front Neurosci. (2018) 12:637. 10.3389/fnins.2018.00637 30294251 PMC6158386

[56] Huang TL Lee CT Liu YL. Serum brain-derived neurotrophic factor levels in patients with major depression: effects of antidepressants. J Psychiatric Res. (2008) 42:521–5. 10.1016/j.jpsychires.2007.05.007 17585940

[57] Brunoni AR Lopes M Fregni F. A systematic review and meta-analysis of clinical studies on major depression and BDNF levels: implications for the role of neuroplasticity in depression. Int J Neuropsychopharmacol. (2008) 11:1169–80. 10.1017/S1461145708009309 18752720

[58] Feingold JH Drossman DA. Deconstructing stigma as a barrier to treating DGBI: lessons for clinicians. Neurogastroenterol Motil. (2021) 33:e14080. 10.1111/nmo.14080 33484225 PMC8091160

[59] Ruddy J. Review article: the patients' experience with irritable bowel syndrome and their search for education and support. Aliment Pharmacol Ther. (2021) 54(Suppl. 1):S44–52. 10.1111/apt.16643 34927755

